# Juquitiba-like Hantavirus from 2 Nonrelated Rodent Species, Uruguay

**DOI:** 10.3201/eid1409.080455

**Published:** 2008-09

**Authors:** Adriana Delfraro, Lorena Tomé, Guillermo D’Elía, Mario Clara, Federico Achával, José C. Russi, Juan R. Arbiza Rodonz

**Affiliations:** Universidad de la República, Montevideo, Uruguay (A. Delfraro, L. Tomé, M. Clara, F. Achával, J. Arbiza Rodonz); Universidad de Concepción, Concepción, Chile (G. D´Elía); Ministerio de Salud Pública, Montevideo (J.C. Russi [retired])

**Keywords:** Hantavirus, Oligoryzomys, Oxymycterus, Sigmodontinae, Uruguay, dispatch

## Abstract

Serologic and genetic analyses indicate that a Juquitiba-like hantavirus circulates in Maldonado, Uruguay. This virus is carried by 2 rodent species, *Oligoryzomys nigripes* and *Oxymycterus nasutus*. The same hantavirus in 2 nonrelated species can be explained by a spillover infection or a host-switching event.

Most hantaviruses (family *Bunyaviridae*) are hosted by 1 or a few closely related rodent species, including rodents of the family Cricetidae, subfamily Sigmodontinae (*1*). Sigmodontine rodents are highly diverse and comprise ≈84 genera (*2*); several hantavirus lineages associated with species belonging to 3 of its tribes—Akodontini, Oryzomyini, and Phyllotini—have been characterized (*3*–*5*).

In Uruguay, 2 closely related hantaviruses—Lechiguanas and Andes Central Plata—cause hantavirus pulmonary syndrome (HPS). Each of these viruses is carried by the yellow pigmy rice rat (*Oligoryzomys flavescens*) (*6*). We report an HPS case in Maldonado, Uruguay, and describe the serologic and genetic analysis carried out on rodents captured at the presumed site of infection.

## The Study

A case of HPS was diagnosed at the Uruguayan Department of Maldonado in February 2005. The patient worked and lived in a greenhouse near Punta Ballena (34°55′S, 55°3′W).

To determine the source of the patient’s infection, during March 12–15 and December 10–11, 2005, small mammals were trapped near where the patient had lived or worked during the 6 weeks before the onset of symptoms and in nearby habitats. Established biosafety guidelines were followed (*7*). Each specimen was identified in the field by using external characteristics. Taxonomic identification of seropositive rodents was corroborated by analyzing skull characteristics and by comparing mitochondrial DNA sequences with sequences available in GenBank. Voucher specimens were deposited at the Mammal Collection of the Facultad de Ciencias, Universidad de la República, Montevideo, Uruguay.

In March 2005, during 1,100 trap nights, 133 rodents belonging to the subfamilies Sigmodontinae (family Cricetidae) and Murinae (family Muridae) were collected (trap success rate 12.1%). Nine months later, in December, 45 rodents belonging to the same subfamilies were collected (trap success rate 8.2%) (Table 1). Immunoglobulin G antibodies to Maciel hantavirus were detected by ELISA (*6*) in 5 rodents (collected in March): 4 long-nosed mice (genus *Oxymycterus*), 6% seroprevalence, and 1 black-footed pigmy rice rat (*Oligoryzomys nigripes)*, 3.2% seroprevalence. (Maciel antigen for the ELISAs was kindly provided by S. Levis, Instituto Nacional de Enfermedades Virales Humanas, Argentina.) A serum dilution was considered positive if optical density was >0.2 U after adjustment. A serum titer >400 was considered positive. Titration showed that all samples had titers >6,400.

**Table 1:** Rodent species captured during 2 trapping expeditions, Maldonado, Uruguay, 2005

Expedition date	Rodent species captured, no. (%)						
	*Oxymycterus* spp.	*Oligoryzomys nigripes*	*Scapteromys tumidus*	*Oligoryzomys flavescens*	*Akodon azarae*	*Necromys obscurus*	*Rattus rattus*
Mar	66 (49.6)	31 (23.3)	14 (10.5)	14 (10.5)	6 (4.5)	1 (0.8)	1 (0.8)
Dec	23 (51.1)	2 (4.4)	5 (11.1)	6 (13.3)	7 (15.6)	0 (0)	2 (4.4)

Molecular methods corroborated the field identification of the 1 black-footed pygmy rice rat and assigned the 4 long-nosed mice to 1 of the 2 species of *Oxymycterus* inhabiting Uruguay: *O. nasutus*. All 5 specimens (3 male, 2 female) were captured in areas of human disturbance: road borders, shrublands, and artificial pine woods (*Acacia* spp. or *Eucalyptus* spp.).

Total RNA was extracted from lung tissue of seropositive rodents and from serum of the HPS patient (*6*). Nested or seminested reverse transcription–PCRs (RT-PCRs) were performed for partial small (S) and medium (M) segments (*8*,*9*). PCR products were sequenced with the same primers used in the RT-PCRs. Viral RNA was detected in 4 of 5 rodents that were seropositive for M or S segments; material was not available for viral RNA testing for 1 *O. nasutus* mouse. RT-PCR amplification attempts on the patient’s serum failed for both segments.

**Figure 1:**
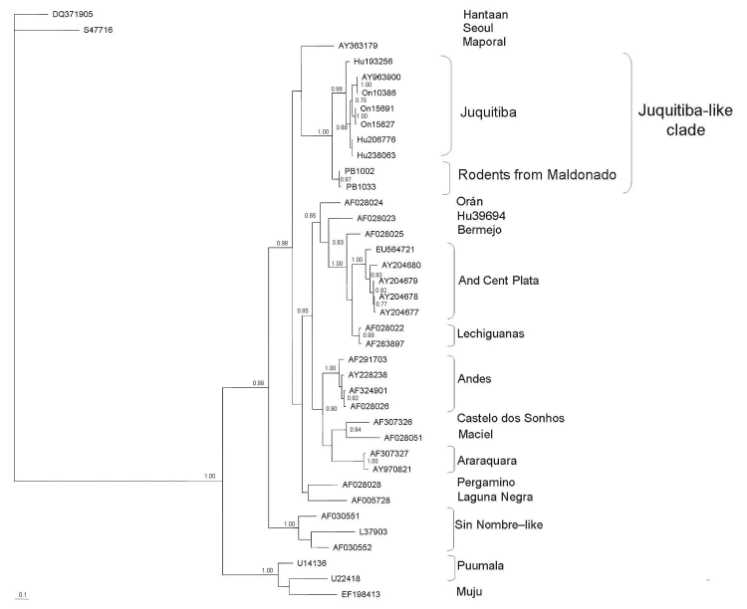
Majority-rule consensus tree obtained in the Bayesian analysis of sequences of the medium segment of Juquitiba-like hantavirus isolated from 2 nonrelated rodent species. Posterior probabilities >0.80 are shown at the nodes. Alignment and editing of nucleotide sequences were conducted by using BioEdit v7.0.9.0 (www.mbio.ncsu.edu/BioEdit/BioEdit.html). Sequences of Seoul and Hantaan hantaviruses were used as outgroup. Estimation of the suitable model of nucleotide substitution and phylogenetic analyses were carried out by using Modelgenerator (http://bioinf.may.ie/software/modelgenerator), MrBayes v3.1.2 (Bayesian analysis; http://mrbayes.csit.fsu.edu), and PAUP* 4.0b10 (maximum-parsimony analysis; http://paup.csit.fsu.edu). Bayesian analyses were conducted under the general time reversible + gamma + proportion invariant model. Two runs of 4 chains each (1 cold, 3 heated, temperature 0.20) were run for 3 million generations; trees were sampled every 100 generations. Convergence was assessed by using the average standard deviation in partition frequency values across independent analyses with a threshold value of 0.01; burn-in was set to 25%. Seropositive specimens from Uruguay are as follows: PB1033 (black-footed pigmy rice rat, *Oligoryzomys nigripes*) and PB1002 (long-nosed mouse, *Oxymycterus nasutus*), GenBank accession nos. EU564726 and EU564725, respectively. Analyzed hantavirus sequences are Hantaan, (DQ371905), Seoul (SA7716), Juquitiba (AY963900, On10386, On15691, On15827, Hu206776, Hu238063, Hu193256), Maporal (AY363179), Andes Central Plata (AY204678, AY204677, AY204679, AY204680, EU564721), Lechiguanas (AF028022, AF283897), Bermejo (AF028025), Hu39694 (AF028023), Orán (AF028024), Andes (AF291703, AF324901, AF028026, AY228238), Castelo dos Sonhos (AF307326), Maciel (AF028051), Araraquara (AF307327, AY970821), Pergamino (AF028028), Laguna Negra (AF005728), Sin Nombre–like (L37903, AF030552, AF030551), Puumala (U14136, U22418), and Muju (EF198413). Scale bar indicates expected changes per site.

**Figure 2:**
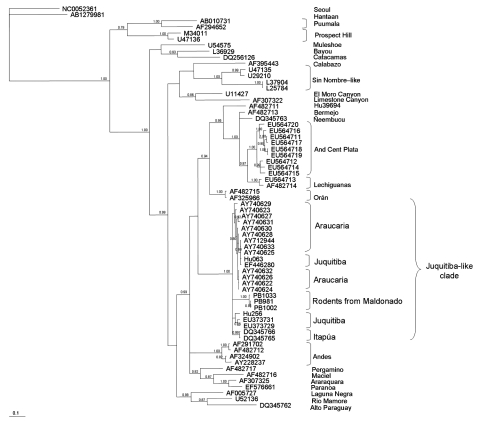
Majority-rule consensus tree obtained in the Bayesian analysis of sequences of the small segment of Juquitiba-like hantavirus isolated from 2 nonrelated rodent species. Posterior probabilities >0.80 are shown at the nodes. Analyses were performed as described in Figure 1. Seropositive specimens from Uruguay are as follows: PB1033 (black-footed pigmy rice rat, *Oligoryzomys nigripes*), PB981 and PB1002 (long-nosed mouse, *Oxymycterus nasutus*), GenBank accession nos. EU564724, EU564722, EU564723, respectively. Analyzed hantavirus sequences are Seoul (NC0052361), Hantaan (AB1279981), Laguna Negra (AF005727), Rio Mamore (U52136), Alto Paraguay (DQ345762), Andes Central Plata (EU564711, EU564717, EU564718, EU564719, EU564716, EU564720, EU564715), Lechiguanas (EU564713, AF482714), Bermejo (AF482713), Ñeembucu (DQ345763), Hu39694 (AF482711), Orán (AF482715, AF325966), Araucaria (AY740627, AY712944, AY740631, AY740633, AY740625, AY740628, AY740632, AY7406261, AY7406221, AY7406241, AY740630, AY740629, AY740623), Juquitiba (Hu063, EF446280, Hu256, EU373729, EU373731), Itapúa (DQ345766, DQ345765), Andes (AF291702, AF482712, AF324902, AY228237), Maciel (AF482716), Araraquara (AF307325), Paranoa (EF576661), Pergamino (AF482717), Sin Nombre–like (U47135, U29210, L37904, L25784), Calabazo (AF395443); El Moro Canyon (U11427), Limestone Canyon (AF307322), Bayou (L36929), Catacamas (DQ256126), Muleshoe (U54575), Puumala (AB010731, AF294652), and Prospect Hill (M34011, U47136). Scale bar indicates expected changes per site.

M segment amplicons (G2 glycoprotein–encoding region) were 735 bp; S segment amplicons were 416 bp. No sequence was obtained from sample PB1011. Similarly, the sequence of the M segment from sample PB981 was not long enough to be included in the phylogenetic analyses. For sequence comparison and phylogenetic analyses, sequences of representative New and Old World hantaviruses were obtained from GenBank. Phylogenetic analyses were conducted by using Bayesian inference and maximum parsimony (Figures 1, 2).

Bayesian analysis based on partial M segment sequences (Figure 1) showed that the sequences retrieved from 1 black-footed pygmy rice rat (PB1033) and 1 long-nosed mouse (PB1002) form a strongly supported clade (posterior probability [PP] 0.97). The Uruguay clade is sister to a clade (PP 1.00) formed by Juquitiba (JUQ) hantaviruses retrieved from black-footed pigmy rice rats (prefix On and AY963900) in Brazil and human HPS case-patients (Hu) in Brazil. We refer to this strongly supported (PP 1.00) clade formed by Brazil and Uruguay sequences as the JUQ-like clade. This clade is sister to Maporal hantavirus. Maximum-parsimony analysis (available from A. Delfraro upon request) also recovers the JUQ-like clade (bootstrap support 100%).

Phylogenetic analysis of S-segment sequences showed a broader taxonomic coverage than that of the M segment (Figure 2). The clade containing the viruses from Uruguay is part of a larger and strongly supported (PP 1.00) clade also formed by JUQ hantaviruses from Argentina (EU373731 from an HPS case-patient; EU373729 from a black-footed pygmy rice rat) and Brazil (Hu256 and Hu063 from HPS case-patients; EF446280 from a black-footed pygmy rice rat), as well as by Araucaria viruses (from HPS case-patients from Brazil) and Itapúa viruses from southern Paraguay, a viral lineage also detected in black-footed pygmy rice rats (DQ345765-66). (Sequences from Brazil JUQ hantaviruses, isolated from *O. nigripes* and HPS case-patients, were kindly provided by S. Levis, Instituto Nacional de Enfermedades Virales Humanas, Argentina.)

Remarkably, JUQ and Araucaria viruses do not form monophyletic groups. The JUQ-like clade is sister to a clade formed by Hu39694, Ñeembucu, Bermejo, Andes Central Plata, Lechiguanas, and Oran; however, this relationship is weakly supported. Maximum-parsimony analysis (not shown) also recovers a strongly supported JUQ-like clade (bootstrap support 100%).

Sequence comparison for M and S segments showed that hantaviruses that cluster in the JUQ-like clade, including the Uruguay samples, form a homogeneous group that shows a high identity percentage at the nucleotide and amino acid levels. Under the general time reversible + gamma + proportion invariant model, the most similar lineages to the JUQ-like viruses are Hu39694 for the M segment and Oran for the S segment (Table 2).

**Table 2:** Sequence comparison for medium and small segments of JUQ-like clade*

Identity or distance	Segment size	
	Medium	Small
Percentage identity†		
Nucleotide	92.6	95.4
Amino acid	98.9	99.7
Mean distance‡		
HU39694	0.451	0.475
Maporal	0.567	NA
Orán	0.631	0.326
Maciel	0.662	0.704
Pergamino	0.707	0.364
Castelo dos Sonhos	0.782	NA
Andes	0.820	0.434
Lechiguan	0.840	0.449
Bermejo	0.886	0.404
Araraquara	NA	0.437
JUQ-like mean distance	0.093	0.039

*Parameters used in the analyses were medium segment, gamma shape 0.29, proportion of invariable sites 0.18; small segment, gamma shape 0.59, proportion of invariable sites 0.38. Sequence comparisons were conducted by using MEGA version 4.0 (www.megasoftware.net) and Modelgenerator (http://bioinf.may.ie/software/modelgenerator). JUQ, Juquitiba; NA, not available. †Identity for the JUQ-like clade. ‡Distance between the JUQ-like clade and several South American hantavirus lineages, under the general time reversible + gamma + proportion invariant model.

## Conclusions

Genetic and phylogenetic analyses showed that in Maldonado, *O. nigripes* and *O. nasutus* carried the same type of hantavirus. Moreover, the viruses from Uruguay form a well-supported clade with JUQ, Araucaria, and Itapúa viruses from HPS case-patients and black-footed pigmy rice rats (*O. nigripes*) from Argentina, Brazil, and Paraguay. In view of the high sequence similarity and the well-supported phylogenetic relationship, we propose that all of these viruses should be considered as JUQ-like hantaviruses. Additional studies will clarify whether all the viruses in the JUQ-like clade represent 1 viral type.

No *Oxymycterus* spp. has been previously reported as being a hantavirus reservoir host. Antibodies to hantavirus in Uruguay *Oxymycterus* spp. may be interpreted as a secondary infection (spillover) (*10*). Interestingly, in the trapping area, the long-nosed mice are the most abundant rodent species, and the seroprevalence of hantavirus among them (6%) is higher than that previously reported in Uruguay (*6*). Black-footed pygmy rice rats are the second most abundant rodent captured; however, the seroprevalence of hantavirus among them (3.2%) is lower.

The presence of the same or similar hantavirus in 2 different rodent species may represent an event of host switching (*5*,*11*–*14*). Hantaviruses detected in Maldonado are similar, although not identical, and are carried by 2 distantly related rodent species that belong to different tribes, Akodontini (*Oxymycterus*) and Oryzomyini (*Oligoryzomys*). Further investigation of the *O. nasutus–*derived hantaviruses is needed to elucidate whether long-nosed mice are true reservoirs or only incidental hosts. Clarification may come from complete sequencing of M and S segments, viral isolation attempts, and new capture expeditions to look for more seropositive long-nosed mice in different areas of Uruguay.
